# Impact of Ultraviolet Radiation on the Aging Properties of SBS-Modified Asphalt Binders

**DOI:** 10.3390/polym11071111

**Published:** 2019-07-01

**Authors:** Huanan Yu, Xianping Bai, Guoping Qian, Hui Wei, Xiangbing Gong, Jiao Jin, Zhijie Li

**Affiliations:** 1 National Engineering Laboratory for Highway Maintenance Technology, School of Traffic and Transportation Engineering, Changsha University of Science & Technology, Changsha 410114, China; 2 School of Traffic and Transportation Engineering, Changsha University of Science & Technology, Changsha 410114, China; 3 Guizhou Transportation Planning Survey & Design Academe Co., Ltd., Guizhou 550000, China

**Keywords:** SBS-modified asphalt binder, UV aging, rheological properties, functional group, cracking

## Abstract

Styrene Butadiene Styrene (SBS) polymer-modified asphalt binders have become widely used in asphalt pavement because of their advantages in high- and low-temperature performance and fatigue resistance. Asphalt pavement is inevitably exposed to sunlight and ultraviolet (UV) radiation during its construction and service life. However, consideration of the aging effect of UV radiation is still limited in current pavement design and evaluation systems. In order to evaluate the impact of UV radiation on the aging properties of SBS-modified asphalt binders, UV aging tests were performed on Rolling Thin Film Oven Test (RTFOT)-aged samples with different UV radiation intensities and aging times. Sixteen different groups of tests were conducted to compare the rheological properties and functional group characteristics of SBS-modified asphalt binders. Dynamic Shear Rheometer (DSR), Bending Beam Rheometer (BBR), FTIR, and SEM tests were conducted to evaluate the aging mechanisms in various UV aging conditions. The results found that UV radiation seriously destroys the network structure formed by the cross-linking effect in SBS-modified asphalt binders, which aggravates the degradation of SBS and results in a great change of rheological properties after UV aging. The nature of SBS-modified asphalt binder aging resulted from the degradation of SBS and the changes of asphalt binder base composition, which lead to the transformation of colloidal structure and the deterioration of asphalt binder performance. The tests also found that continuous UV radiation can increase shrinkage stress in the asphalt binder surface and leads to surface cracking of the asphalt binder.

## 1. Introduction

Asphalt pavements are widely used in the construction of roads because of their advantages of having a smooth surface, seamlessness, lower driving noise, easy maintenance, and recyclability [1,2]. Among all asphalt binder modification types, Styrene Butadiene Styrene (SBS)-modified asphalt binders are widely used in pavement areas because of their excellent performance under high and low temperature and their fatigue resistance, and they have been applied in a huge percentage of global asphalt pavement construction projects [3].

Normally, asphalt binder aging is characterized into short-term aging and long-term aging. The short-term aging typically occurs at the mixing and paving process, and the long-term aging occurs during the whole service life. Researches have pointed out that a wide range of factors impact the properties of the polymer/asphalt system [4,5]. As asphalt pavements are inevitably exposed to UV radiation, water, and thermal and other environments during their whole service life, the long-term aging is a combined impact of various factors. Consequently, different countries have developed several standardized accelerated aging methods in laboratory to simulate the in-field aging process. However, very little consideration has been given to the impact of UV radiation on aging.

Researchers have found that the impact of UV radiation has led to the aging of asphalt pavements, which results in the deterioration of road performance and the reduction of road service life [6]. Furthermore, UV radiation from sunlight has become more and more intense because of global warming and environmental changes, and thus the impact of UV aging on asphalt pavements is becoming more and more serious.

Some researchers pointed out that UV radiation might only impact the upper layers of the asphalt pavement surfacing, and the influence of UV radiation on asphalt aging has always been ignored in laboratory simulations of aging [7]. However, Durrieu [8] pointed out that the impact of UV radiation on the aging of asphalt pavements cannot be ignored, and the research found that the aging of asphalt samples under 10 hours of UV radiation in the lab was equal to the same level of Rolling Thin Film Oven Test (RTFOT) and Pressure Aging Vessel (PAV) aging, or aging equal to one year of service in field.

In order to quantify the rate and degree of UV aging of asphalt binders, Zheng et al. [9] established the nonlinear equation of the performance attenuation of asphalt binders after UV aging by using three indexes of penetration, viscosity, and ductility, respectively. Wang et al. [10] studied the effect of UV aging on the aggregated state of asphalt binders by various test methods, and found that UV aging reduced the rheological characteristics of asphalt binders, and that asphalt material is transformed from a viscous material to an elastic material under normal temperature. Xiao et al. [11] compared the impacts of long-term thermal and UV aging on foamed Warm Mix Asphalt (WMA) mixtures, and found that UV-aged mixtures had reduced the Indirect Tension Strength (ITS) values and increased dissipated energy compared to other asphalt mixtures. Kemp et al. [12] found that a 5–10 μm-thick layer of harder film forms above the asphalt binder under UV radiation. When the asphalt film thickness is greater than 200 μm, the change of the asphalt binder functional group is obviously decreased.

By means of Dynamic Shear Rheometer (DSR), FTIR, the Indirect Tension Strength (GPC), nuclear magnetic resonance hydrogen spectroscopy (1H-NMR), and other experiments, Ran [13] explored the changes of the macroscopic properties and microstructure of base asphalt binders and SBS-modified asphalt binders under the coupling of heat, light, and water, and, based on the physical and rheological properties, the nonlinear coupled aging rate prediction differential equation and the aromaticity microstructure aging kinetic equation were established. Wei et al. [14] evaluated the aging behavior of SBS-modified asphalt binders under various UV and water conditions, and found that water aggravates the UV aging of asphalt binder material, and the presence of acid or salt worsens UV aging. Zeng et al. [15] found that UV radiation can only transmit within 4.5 μm of the surface layer, but the UV aging depth can reach deeper and increases with the UV radiation time. The research pointed out that the different components of surface-aged asphalt binders diffuse to the lower parts of the asphalt binders, leading to the increasing of aging. Mouillet et al. [16] evaluated the impacts of UV radiation on elastomer-modified asphalt binders through FTIR and Scanning Electron Microscope (SEC) methods, and their results found that the elastomer architecture did not impact the degradation under UV radiation.

Hou et al. [17] pointed out that Fourier transform infrared spectroscopy (FTIR) is able to evaluate the aging characteristics and mechanisms of asphalt binder materials. Hu et al. [18] evaluated the impacts of different wavebands of UV radiation on the aging of asphalt binder materials, and found that UV aging has a great impact on the low-temperature performance of asphalt binders. Zeng et al. [19] pointed out that the effect of temperature on UV aging cannot be ignored, and suggested that the UV aging tests should be performed below 50 °C. Their research also pointed out that the coupling of high temperature and UV radiation accelerated the volatilization rate and oxidation rate of light components.

In recent years, scholars have made certain achievements in the study of asphalt binder UV aging. However, due to the variability of asphalt binder components and the complexity of the UV aging process, the understanding of the mechanism of asphalt binder UV aging is still not clear, and a standard asphalt binder UV aging evaluation system has not been established. In addition, the existing UV aging studies of asphalt binders are mainly focused on the base asphalt, while the studies on polymer-modified asphalt binders are relatively few in number. Therefore, in order to evaluate the macroscopic properties and the microstructure of SBS-modified asphalt binders, this paper conducted rheological tests and FTIR tests to reveal the aging characteristics and mechanisms of SBS-modified asphalt binders under different UV light intensities and time conditions.

## 2. Materials and Methods

### 2.1. Materials

SBS-modified asphalt binders supplied by Hua Te Asphalt Co., Ltd. (Xiamen, China) were used in this research, with an SBS concentration of 4%. The conventional tests were conducted following the procedures of “Standard Test Methods of Bitumen and Bituminous Mixtures for Highway Engineering” (JTG E20-2011) [20]. The test results and specification requirements are shown in Table 1, and it can be seen from that table that the asphalt binders satisfied the specification requirements.

**Figure 1:**
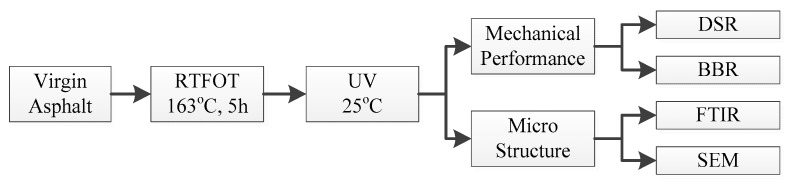
The flowchart of experimental design.

The flow chart of the experimental design is shown in Figure 1. Firstly, the virgin asphalt binder was short-time aged by RTFOT, and then UV aging was conducted on the short-time aged samples. Then, the Dynamic Shear Rheometer (DSR) and Bending Beam Rheometer (BBR) tests were conducted to evaluate the rheological properties of the aged samples, and Fourier Transform Infrared Spectroscopy (FTIR) and Scanning Electron Microscope (SEM) tests were conducted to evaluate the microstructure of the aged samples. Three duplicated samples were prepared for each test.

### 2.2. Aging Procedure

RTFOT short-term aging tests was conducted following the procedure of “Standard Test Methods of Bitumen and Bituminous Mixtures for Highway Engineering” (JTG E20-2011) [20]. UV aging tests were performed in an UV weathering chamber at a wavelength of ultraviolet long-wave (UVA) 365 nm. The steps for UV aging preparation are shown below:

Firstly, we poured around 15 ± 0.1 g of the RTFOT-aged SBS-modified asphalt binder into a 85 × 85 mm silicone paper carton, then placed it in an oven at 160 °C for 3 min, and then took it out to allow it cool down to room temperature. Then, a sample of asphalt binder with film thickness of 2 mm was prepared. Then, the samples were put into a UV radiation (UVA 365 nm; LED) chamber to conduct UV aging, and the UV radiation chamber was set at a temperature of 25 °C to eliminate the impact of temperature changes. The UV radiation tests were conducted at a range from 40 to 160 h, with an interval of 40 h. As the environmental UV radiation intensity between the Class-I and Class-II standards of China is close to 5 mW/cm^2^, and for the consideration of accelerated aging, the UV intensity ranged from 5 to 20 mW/cm^2^, with an interval of 5 mW/cm^2^ [21]. In this research, the naming and UV radiation tests schematic are shown in Table 2.

### 2.3. Dynamic Shear Rheometer (DSR)

The rheological properties (complex modulus and phase angle) of the SBS-modified asphalt binders before and after UV aging with different UV aging times and intensities were investigated by the DSR test. The DSR test instrument was Physica MCR 301 by Anton Paar (Ostfildern, Germany). Temperature sweep tests were conducted under the strain-controlled mode with a constant frequency of 10 rad/s, and the temperature ranged from 40 to 90 °C, with an increment of 2 °C/min. The test specimens were prepared with a set of parallel plates of 25 mm diameter, with a 1 mm gap between each plate according to the AASHTO T315-05 [22].

### 2.4. Bending Beam Rheometer (BBR)

BBR tests were carried on RTFOT- and UV-aged specimens by a Cannon thermoelectric rheometer at two different temperatures of −12 and −18 °C, following the procedures of American Association of State Highway and Transportation Officials (AASHTO) T313-12 [23]. The load and deformation of all specimens at 8, 15, 30, 60, 120, and 240 s were collected, and the stiffness modulus *S* and creep rate *m* were calculated. Three duplicated samples were prepared for each test. The values of *S* and *m* at 60 s were used to characterize the low-temperature properties of SBS-modified asphalt binders with different UV aging degrees.

### 2.5. Fourier Transform Infrared Spectroscopy (FTIR)

**Figure 2:**
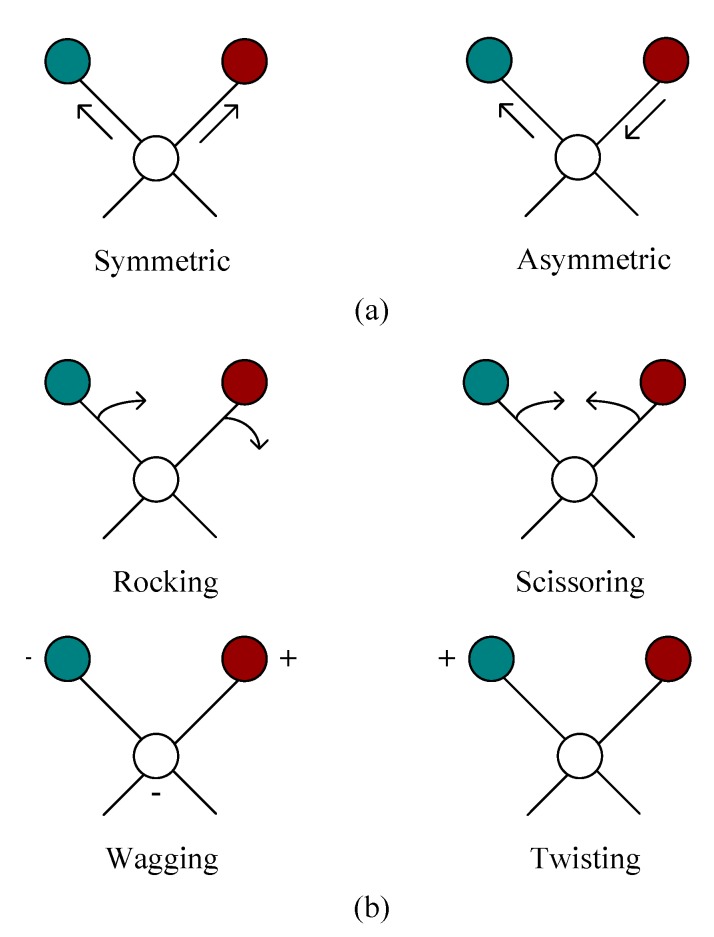
Molecular vibration form: (**a**) Stretching vibration and (**b**) bending vibration.

FTIR is a quantitative and qualitative technique for analyzing the contents of organic components based on the changes of functional groups, which has been demonstrated to be an effective tool to describe the aging characterization of asphalt binders [17,24,25]. The principle of FTIR is that the molecules vibrate when organic compounds absorb infrared light at the corresponding wavelength, and the molecular vibration can be divided into stretching vibration and bending vibration (see Figure 2). Since those two vibrational types have their corresponding absorption peaks in infrared spectra, the existence of functional groups and the contents of components in the molecules can be identified according to the positions and intensities of the absorption peaks.

**Figure 3:**
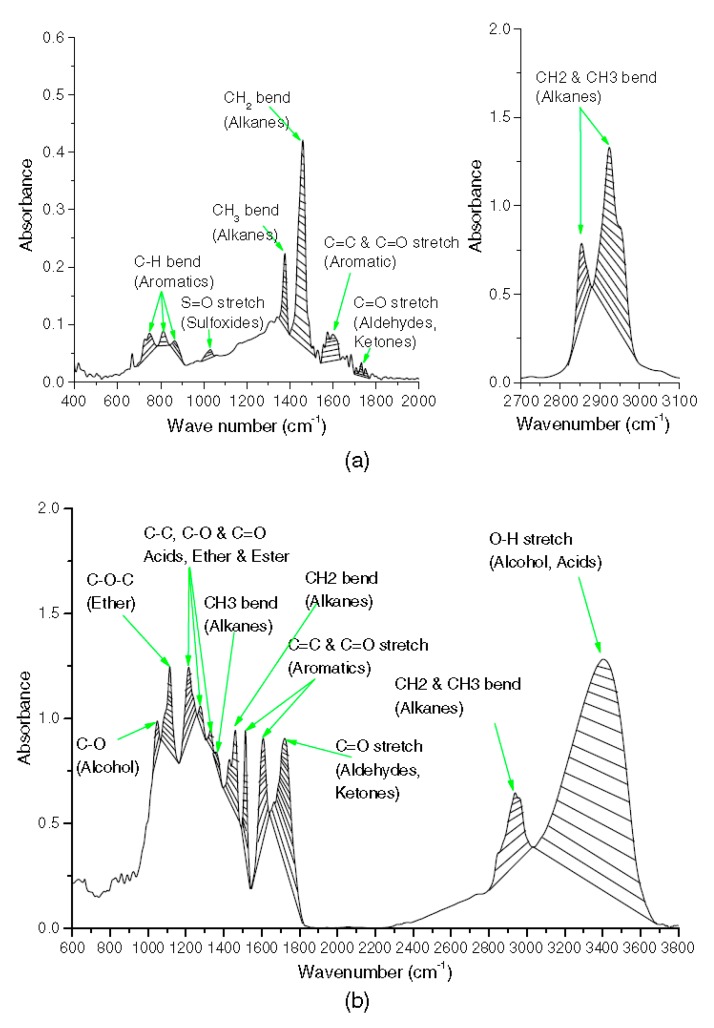
Spectra and functional groups: (**a**) Spectra and functional groups for the asphalt binder; (**b**) spectra and functional groups for the biobinder [27].

In this study, the changes of specific functional groups of asphalt binders with different aging degrees were evaluated by FTIR (Tensor 27 of Bruker manufactured by Germany). The absorbance spectra of asphalt binders were recorded, ranging from 4000 to 400 cm^−1^, and the spectral resolution was 1–0.4 cm^−1^. The main functional group band assignments and positions are summarized in Table 3. Furthermore, the main aging indexes, including carbonyl group (C=O) and sulfoxide group (S=O), were introduced for a more intuitive result [16,26], as shown in Equations (1)–(4):

(1) $$I_{C=O}=\frac{A_{1700}}{A_{1460}+A_{1375}}$$

(2) $$I_{S=O}=\frac{A_{1030}}{A_{1460}+A_{1375}}$$

(3) $$I_{PB}=\frac{A_{966}}{A_{1460}+A_{1375}}$$

(4) $$I_{PS}=\frac{A_{699}}{A_{1460}+A_{1375}}$$

where *I_C=O_* is the aging index of the carbonyl group (C=O), *I_S=O_* is the aging index of the sulfoxide group (S=O), *I_PB_* is the aging index of butadiene (PB), and *I_PS_* is the aging index of styrene (PS). *A* is the area of the corresponding absorption peak. For example, *A*_1700_ refers to the absorption peak at a wavenumber of 1700 cm^−1^. Figure 3 shows a typical FTIR spectra and the functional groups of petroleum asphalt and biobinders [27].

### 2.6. Scanning Electron Microscope (SEM)

The scanning electron microscope (SEM) is an important tool which can be used to observe the micro-morphology and microstructure of materials. The principle is that by emitting high-energy electron beams on a sample, when the electron beams come into contact with the surface of the sample, they will produce information such as secondary electron, scattered back electron, and so on, and the information will be changed from optical signals to electric signals through systematic processing. After the video amplifier is amplified, the image of the sample surface can be formed on the screen of the picture tube. In this paper, the aging asphalt binder samples were scanned by SEM under different conditions to observe the surface cracking characteristics of the asphalt binder samples after UV aging.

## 3. Results

### 3.1. High-Temperature Performance

**Figure 4:**
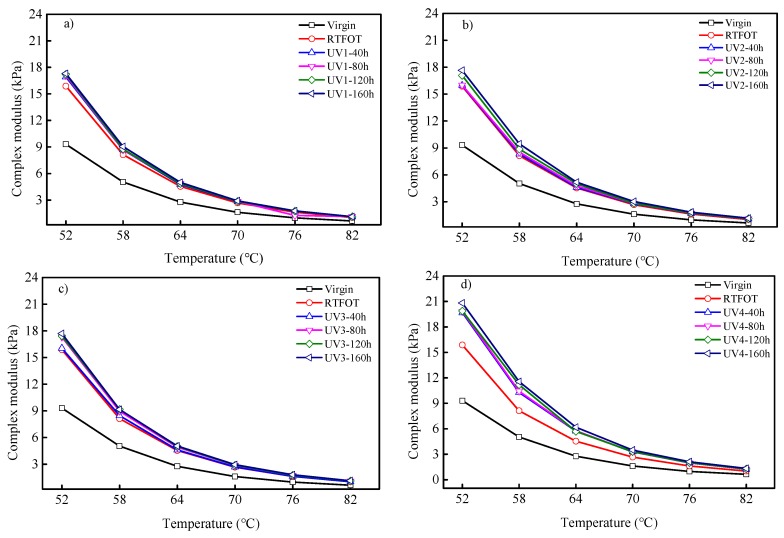
Complex modulus of SBS-modified asphalt binders under different UV intensities: (**a**) UV = 5 mW/cm^2^; (**b**) UV = 10 mW/cm^2^; (**c**) UV = 15 mW/cm^2^; (**d**) UV = 20 mW/cm^2^.

**Figure 5:**
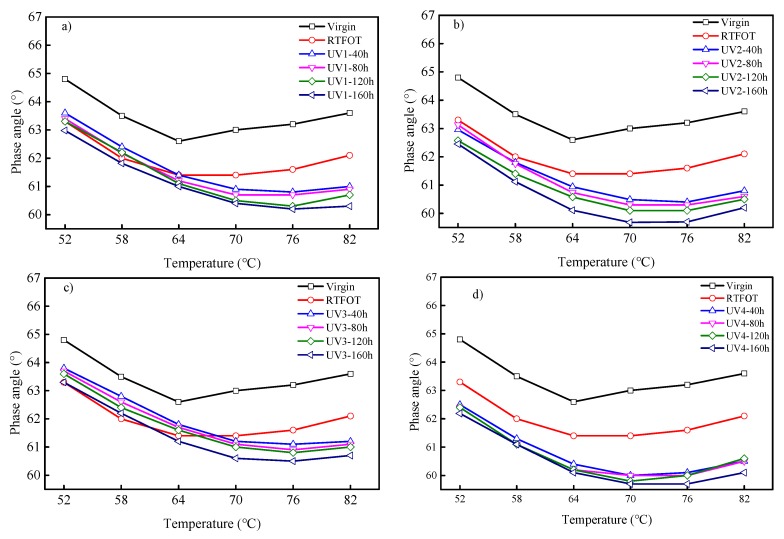
Phase angle of SBS-modified asphalt binders under different UV intensities: (**a**) UV = 5 mW/cm^2^; (**b**) UV = 10 mW/cm^2^; (**c**) UV = 15 mW/cm^2^; (**d**) UV = 20 mW/cm^2^.

The temperature sweep data from the DSR testing was used to obtain the indexes of the complex modulus and phase angle, as shown in Figure 4 and Figure 5. It was concluded from Figure 4 that, together with the UV aging time or UV intensity increment, the complex modulus of SBS-modified asphalt binders also increased. With the prolongation of aging time and the increase of UV intensity, asphalt binder aging became worse, and the light component of the asphalt binder was transferred to the recombination fraction. In addition, the more serious the aging, the more volatile the light components. After aging, the viscosity of the asphalt binder decreased, the elastic component increased, and the asphaltene content increased, which show that the asphalt binder hardens, so the composite modulus gradually becomes larger. It can be observed that SBS-modified asphalt aging kept approximately the same complex modulus under the same aging time when the UV intensity was 15 mW/cm^2^ or less. However, when the UV intensity was greater than 15 mW/cm^2^, the complex modulus was greatly increased. In addition, the curves have different slopes, indicating different temperature susceptibilities between virgin, RTFOT-aged, and UV-aged asphalt.

The phase angle represents the ratio of viscosity to elastic composition of the asphalt binder. The smaller the phase angle, the greater the proportion of the elastic component. It can be seen from Figure 5 that the phase angle of SBS-modified asphalt binders gradually decreases with increasing test temperature at the beginning, and then increases regardless of aging type and aging time, showing different characteristics to virgin asphalt binders. Generally speaking, with the increase of temperature, asphalt binders will gradually change from viscoelastic to viscosity properties, and the phase angle increases gradually. The reduction of phase angle at the beginning is mainly attributed to the existence of SBS. At a certain temperature, SBS has high elasticity and fatigue resistance. When the temperature continues to rise, the polystyrene phase gradually softens and flows to make the SBS plastic. Under the same conditions, the longer the aging time or the greater the UV intensity is, the smaller the phase angle and the greater the level of asphalt binder aging is. Compared with the original asphalt binder and the short-term aging asphalt binder, the turning point temperature of the UV-aging asphalt binder decreases first and then increases, which is due to the serious destruction of the network structure formed by the crosslinking effect in the SB-modified asphalt binder by UV radiation, and the aggravated degradation of SBS. Regardless of UV intensity and aging time, the turning point of the phase angle of SBS-modified asphalt binders was approximately 70 °C, which was about 6 °C higher than that of the virgin asphalt binder and the one after short-term aging.

### 3.2. Low-Temperature Performance

**Figure 6:**
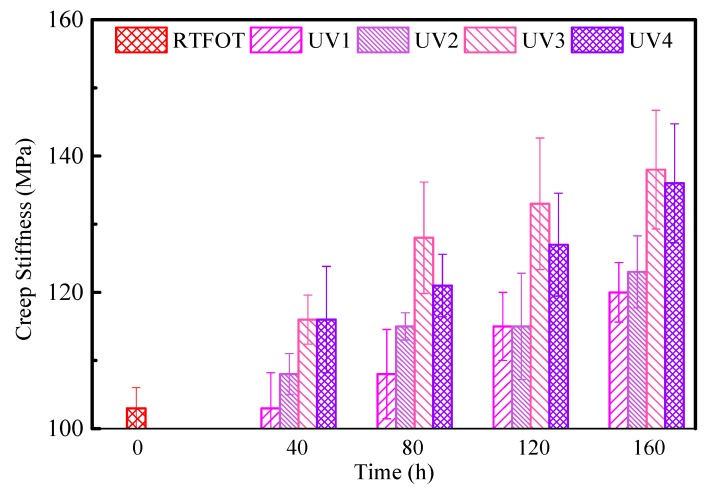
Creep stiffness (*S* value) of SBS-modified asphalt binders under UV and Rolling Thin Film Oven Test (RTFOT) aging.

**Figure 7:**
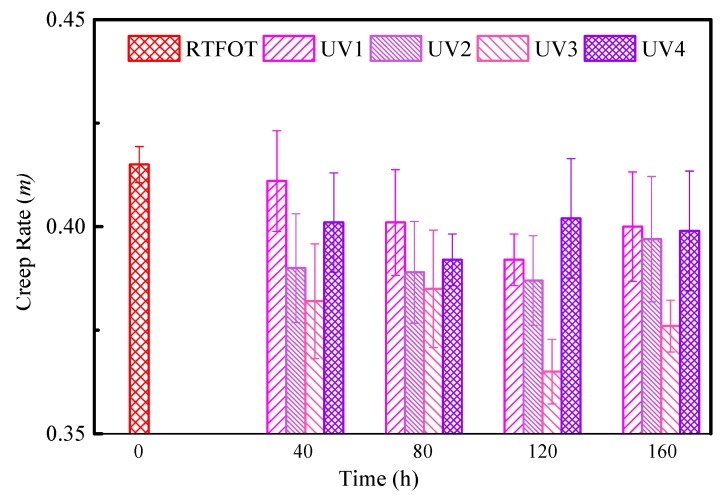
Creep rate (*m* value) of SBS-modified asphalt binders under UV and RTFOT aging.

Strategic Highway Research Program (SHRP) demonstrated that asphalt binder properties greatly impact the low temperatures performance of asphalt mixture [29]. According to the mechanism of low-temperature cracking of asphalt pavement, low-temperature rheology is the most important factor affecting low-temperature cracking of asphalt pavement [30]. Therefore, the aging behavior of asphalt binders can be theoretically evaluated from the perspective of the rheological properties of asphalt binders at low temperature. The test results show that at −18 °C, the SBS-modified asphalt binders with different aging conditions have *S* > 300 MPa and *m* < 0.3. This indicates that the low-temperature performance of SBS-modified asphalt binders has failed at −18 °C, according to the specifications. Therefore, only the BBR test results of asphalt binder samples at −12 °C are listed in this paper, as shown in Figure 6 and Figure 7. The stiffness modulus *S* basically increases with the increase of aging time or UV radiation intensity. However, when the UV radiation intensity reaches a certain level, the stiffness modulus decreases as the strength increases. The creep rate *m* of UV-aged samples shows a slight degree of a decreasing trend, however, the trend is not significant. The reason is mainly due to the fact that UV only affects the surfaces of asphalt binders, and the aging part of the asphalt binder distribution in the beam is different.

### 3.3. Functional Group Change

**Figure 8:**
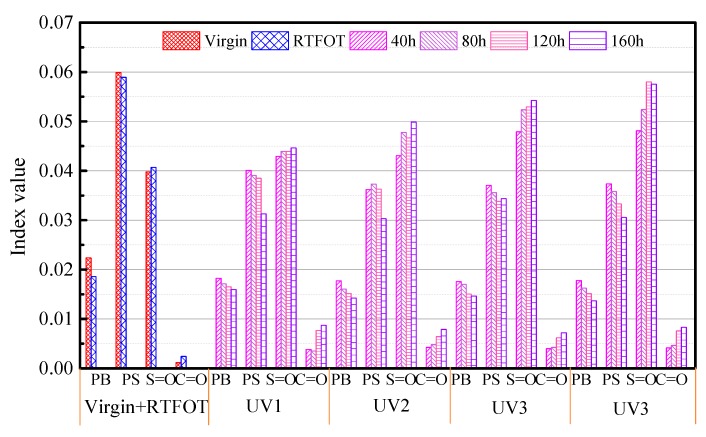
Aging indices of different functional groups in SBS-modified asphalt binders.

Due to the influences of coating thickness, environmental noise, instrument, and background interference on the infrared spectrum test, the spectrum will drift and tilt to a certain extent, so it is necessary to optimize the processing of the original spectral data [31]. In this paper, the normalized preprocessing method is used to eliminate the adverse effects of interference information on the test results and to enhance the differences between spectra. The corresponding functional group aging index is shown in Figure 8.

It can be seen that, after UV aging, the intensities of the stretching vibration absorption peaks of C=O at 1700 cm^−1^ and S=O at 1030 cm^−1^ fluctuate slightly, but the amplitudes are not large. This is due to the C–O oxygenation to C=O and the C–O and C–S breakage to produce S=O during asphalt binder aging. Studies have shown that the degree of aging can be characterized by the *I_C=O_* and *I_S=O_* indices. However, the amplitudes of the C=O and S=O bands in the spectrogram caused by the aging effect are too weak to calculate the peak area accurately. In addition, as the content of S in asphalt binders is fixed, the *I_C=O_* and *I_S=O_* indices are inappropriate to represent the degree of asphalt binder aging.

The symmetric and asymmetric bending vibration absorption peaks of methyl and methylene at 1460 and 1375 cm^−1^, respectively, are the most stable in the spectrum, and therefore, they are often used as the reference bands for quantitative analysis of infrared spectra. The absorption peaks of virgin and SBS-modified asphalt binders at 1460 and 1375 cm^−1^ are basically the same as those of short-term aging asphalt binders. However, after UV aging, the absorption peaks of these two sites increase slightly, and the amplitudes of absorption spectra increase with the increase of UV aging time. This is mainly due to the destruction of C=C bonds of unsaturated olefins in asphalt binders irradiated by UV radiation, and the formation of C–H bonds. The absorption peaks at 966 and 699 cm^−1^ are the differences between SBS-modified asphalt binders and base asphalt binders. The peak at 966 cm^−1^ represents the bending vibration absorption peak of PB C=C in SBS, and the 699 cm^−1^ peak represents the bending vibration peak of PS C–H in SBS. Compared with the virgin asphalt binder and the short-term aging asphalt binder, the amplitudes of these two bands decrease slightly after UV aging, and the decrease in amplitude became greater with the prolongation of UV aging time. It is believed that this is due to the breakdown of SBS segment structure and the decrease of molecular weight with the increase of aging time and UV radiation intensity, which is the main reason for SBS degradation in the aging process of SBS-modified asphalt binders. The results show that the greater the degree of degradation, the stronger the intermolecular conjugation effect.

### 3.4. Apparent Topography

**Figure 9:**
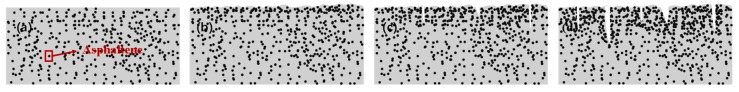
The four stages of asphalt binder base topography change under UV radiation: (**a**) The asphalt binder surface is smooth and flat, (**b**) the asphalt binder surface is wrinkled, (**c**) micro-cracks occur in the asphalt binder surface, and (**d**) the cracks are further extended and expanded to be wider and deeper.

In general, asphalt pavement cracking is mostly in the form of fatigue and low-temperature cracking caused under driving loads and temperature stress, or in the other form of reflective cracking due to the existence of cracks in the base layer. The results of SEM show that UV radiation can also cause top-down cracking of the asphalt binder surfaces at a room temperature of 25 °C. Based on the UV aging level, the asphalt binder surface cracking process can be roughly divided into four stages (as shown in Figure 9).

**Figure 10:**
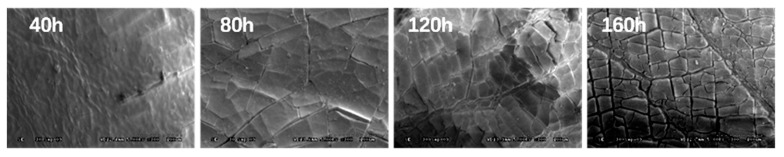
SEM scanning of SBS-modified asphalt binders under different UV radiation times.

Figure 10 shows the apparent topography of SBS-modified asphalt binders under different UV radiation (365 nm, 10 mW/cm^2^) times (magnification 300 times). After 40 h of UV radiation, the surfaces of SBS-modified asphalt binders appeared wrinkled, and there were micro-cracks beginning to be produced. When the UV radiation time reached 80 h, the small cracks extended to become dense cracks on the surfaces of SBS-modified asphalt binders. When the UV radiation time increased to 120 h, the surface cracks further developed and extended, and the surfaces began to fragment. When the UV radiation time reached 160 h, the micro-cracks in the surfaces were further connected and expanded, and the asphalt binder surfaces were completely fragmented and the corners became warped.

According to the cracking criterion of pavements, when the stress of an asphalt pavement reaches the tensile strength of asphalt concrete under load, it will crack. When the stress intensity factor of the crack tip exceeds the fracture toughness of the material, the crack will expand. The cracking criterion of pavement considers that when the stress of an asphalt pavement reaches the tensile strength of asphalt concrete under the action of load, the crack will expand when the stress intensity factor at the crack tip exceeds the fracture toughness of the material [32]. Therefore, the author considers that under UV radiation, the asphalt binder surface will produce a kind of shrinkage stress similar to the temperature stress. In addition, the UV aging will cause the stiffness modulus of the asphalt binders to increase, causing the stress relaxation performance and the ultimate strength of the asphalt binders to decrease. When the shrinkage stress is greater than the limit stress, cracking will occur, and with the increase of the aging level, the crack will expand further.

## 4. Conclusions

In this paper, rheology tests and spectroscopy tests were conducted to investigate the UV aging of asphalt binders. Based on the test results of SBS-modified asphalt binders at different aging states, the following conclusions can be drawn:

(1) UV seriously destroyed the network structure formed by the crosslinking effect in SBS-modified asphalt binders, and the degradation of SBS was aggravated, which resulted in the SBS-modified asphalt binders becoming more homogeneous after aging. The nature of the UV aging includes the change of component content and degradation of SBS, which leads to the transformation of colloidal structure, manifested by the increase of asphaltene, the dynamic stability of resin, and a reduction in the molecular weight of SBS.
(2) The complex modulus of SBS-modified asphalt binders increases continuously, and the phase angle decreases with the increase of aging. Although this means that the rutting resistance is improved, the aging asphalt binders are more rigid and brittle, which can easily lead to the pavement cracking at low temperature. With the increase of temperature, the phase angle decreases at the beginning and then increases, and the turning point temperature of the phase angle increases after UV aging.
(3) Continuous UV radiation can induce a kind of shrinkage stress similar to the temperature stress in the surface asphalt binders at mild constant temperature. When the shrinkage stress exceeds the limit stress of the material, it will lead to the formation of top-down cracks in the surface layers of the asphalt binders.

## Figures and Tables

**Table 1:** Physical properties of Styrene Butadiene Styrene (SBS)-modified asphalt binders.

Item		Value	Specification
Penetration (25 °C, 0.1 mm)		55	40–60
Ductility (5 °C, cm)		34	≥20
R&B temperature, softening point, (°C)		87	≥60
Viscosity (135 °C, Pa·s)		2.1	≤3
Flash point (COC, °C)		318	≥230
RTFOT	Mass loss (%)	0.06	≤±1.0
	Residual penetration ratio (%)	80	≥65
	Residual ductility ratio (%)	21.0	≥15

**Table 2:** UV radiation tests schematic.

Naming (Radiation Intensity)	UV Radiation Time			
UV1 (UV = 5 mW/cm^2^)	40 h	80 h	120 h	160 h
UV2 (UV = 10 mW/cm^2^)	40 h	80 h	120 h	160 h
UV3 (UV = 15 mW/cm^2^)	40 h	80 h	120 h	160 h
UV4 (UV = 20 mW/cm^2^)	40 h	80 h	120 h	160 h

**Table 3:** Functional groups and corresponding wavenumber in infrared spectra [28].

Wavenumber (cm^−1^)	Assignment
2951	ν as CH_3_-aryl
2921	ν as CH_3_, CH_2_
2852	ν s CH_3_, CH_2_
1710	ν C=O
1600	ν C=C
1456	δ as CH_3_, CH_2_
1376	δ s CH_3_
1311	ν SO_2_, or ester groups
1168	ν C–O–C (anhydrides), –S–C
1021	ν O-C=O, C–S=O, ether or ester groups
865	δ CH aromatic (out-of-plane bending)
813	δ CH aromatic (out-of-plane bending)
744	δ CH aromatic (out-of-plane bending)
723	r CH_2_ (rocking CH_2_ groups chains (CH_2_)_n_)
966	ν butadiene(PB)
699	ν styrene (PS)

ν = stretching; δ = bending; s = symmetric; as = asymmetric.
